# Quantitative Oxygen Consumption and Respiratory Activity of Meat Spoiling Bacteria Upon High Oxygen Modified Atmosphere

**DOI:** 10.3389/fmicb.2019.02398

**Published:** 2019-11-08

**Authors:** Sandra Kolbeck, Leonie Reetz, Maik Hilgarth, Rudi F. Vogel

**Affiliations:** Lehrstuhl für Technische Mikrobiologie, Technische Universität München, Freising, Germany

**Keywords:** high oxygen modified atmosphere, meat spoilage, oxygen consumption, respiratory growth, *Brochothrix thermosphacta*, lactic acid bacteria

## Abstract

High oxygen modified atmosphere packaging is a commonly applied method to prolong the minimum shelf life of fresh (red) meats. Upon spoilage, changes of the initial oxygen concentration and microbiome composition can be observed. Thus, we classified the typical representative meat spoiling bacteria *Brochothrix* (*B.*) *thermosphacta* TMW2.2101 and the four lactic acid bacteria (LAB) *Carnobacterium* (*C.*) *divergens* TMW2.1577, *C. maltaromaticum* TMW2.1581, *Leuconostoc* (*L.*) *gelidum* subsp. *gelidum* TMW2.1618, and *L. gelidum* subsp. *gasicomitatum* TMW2.1619 along their oxygen consuming capacity, which can indicate the timeline of microbiome and sensorial changes. All bacteria were grown in a model system employing gas tight glass bottles containing meat simulation media and under modified atmosphere (70% O_2_ and 30% CO_2_). Oxygen concentrations of media and headspaces were monitored over time and the oxygen uptake rate (OUR) was calculated for all species. All bacteria were able to consume dissolved oxygen, with *B. thermosphacta* TMW2.2101 exhibiting a 31-times higher OUR per single cell in 60 h. Furthermore, all strains showed significant growth enhancement in the presence of heme indicating respiratory activity. Comparative genomic and physiological analyses predict the activity of a respiratory chain for all species upon high oxygen atmosphere. An additional cytochrome aa_3_ oxidase is suggested to be responsible for the increased OUR of *B. thermosphacta* TMW2.2101. Furthermore, *B. thermosphacta* TMW2.2101 revealed highest oxidative stress tolerance compared to the other bacteria, facilitating a higher respiratory activity. Coupling of respiration and fermentation via regeneration of NADH can be a competitive advantage for meat spoiling bacteria resulting in a higher cell count and possibly accelerated spoilage. The exhibited highest capacity for oxygen consumption of *B. thermosphacta* compared to LAB *in vitro* also suggests a higher contribution of this bacterium to the change in the atmosphere upon spoilage of MAP meats *in situ*.

## Introduction

Meat spoilage and shelf life extension are one major focus and challenge of the food industry. Spoilage of meat products is caused by growth of specific spoilage organisms, found as initial contaminants on fresh packaged meat. Such microorganisms have been identified as Gram-negative *Pseudomonas* species, representatives of the family *Enterobacterales*, Gram-positive lactic acid bacteria (LAB), *Brochothrix* (*B.*) *thermosphacta* (*Listeriaceae*), *Aeromonas*, and *Shewanella putrefaciens* (Lambert et al., 1991; Borch et al., 1996; Lambropoulou et al., 1996; Carlo et al., 2000; Ercolini et al., 2006; Doulgeraki et al., 2012).

Modified atmosphere packaging (MAP) is one common method to suppress meat spoilers and prolong the shelf-life of fresh meats. Especially red meats, e.g., beef but also white meats such as chicken are offered in packages with high oxygen atmosphere (70% O_2_/30% CO_2_) (Rossaint et al., 2014). One major function of oxygen in MAP red meats is maintaining oxymyoglobin levels responsible for the desired red color by avoiding oxidation to metmyoglobin (Lambert et al., 1991; Borch et al., 1996; Gill, 1996; Seideman et al., 2007). Furthermore, oxygen suppresses the cell growth of strict anaerobic pathogenic bacteria such as *Clostridium botulinum* and *Clostridium perfringens* (Lambert et al., 1991). The other protective gas added to meat packages is carbon dioxide. In concentrations of 20–30%, carbon dioxide inhibits Gram-negative microorganisms, e.g., *Pseudomonas* species (King and Nagel, 1967; Gill and Tan, 1979). It can also induce longer lag-phases of meat-spoiling bacteria thus delaying the onset of spoilage (Gill and Tan, 1980). Hence, the synergistic effect of both protective gases is reported to enable a 7–10 days shelf life extension of meat (Clark and Lentz, 1973; Vihavainen and Björkroth, 2007).

However, many food-spoiling LAB are facultatively aerobic and quite resistant to carbon dioxide (Rossaint et al., 2014). This contributes to the fact that LAB can be found as main spoilers on high oxygen packaged meat (Shaw and Nicol, 1969; Esmer et al., 2011; Rossaint et al., 2014; Hilgarth et al., 2018), e.g., the LAB *Carnobacterium* (*C.*) *divergens* and *Leuconostoc* (*L.*) *gelidum* subspecies are known to induce meat greening by hydrogen peroxide production (Borch and Molin, 1989; Vihavainen and Björkroth, 2007). High oxygen concentrations have been reported to promote the cell growth of facultative aerobic bacteria as demonstrated for *B. thermosphacta* (Newton et al., 1977; Silliker et al., 1977; Rossaint et al., 2014) and induce the production of undesired spoilage compounds, e.g., iso-butyric acid, iso-valeric acids, acetoin, 2-heptanone, and 2-hexanone (Blickstad and Molin, 1984; Borch et al., 1996; Ercolini et al., 2006; Dainty and Hibbard, 2008).

During storage, oxygen concentrations in meat packages are not static, but decrease due to biochemical activity of the initial microbiota (Kennedy et al., 2004; Koutsoumanis et al., 2008; Esmer et al., 2011; Hilgarth et al., 2018). Upon oxygen decrease, a microbial switch to a putrid consortium can be detected, resulting in the production of volatile components causing off flavors (Hilgarth et al., 2018). Thus, oxygen consumption in meat packages is directly linked to rejectionable meat spoilage. As knowledge to the contribution of different meat spoiling bacteria on oxygen consumption is very limited, the aim of this study was to characterize typical representative species of the spoilage microbiota along their oxygen consuming capacity.

## Materials and Methods

### Bacterial Strains

Five spoilage-associated bacteria from the TMW strain collection (*B. thermosphacta* TMW2.2101, *C. divergens* TMW2.1577, *C. maltaromaticum* TMW2.1581, *L. gelidum* subsp. *gelidum* TMW2.1618, and *L. gelidum* subsp. *gasicomitatum* TMW2.1619) were selected in order to characterize their oxygen consuming capacity under high-oxygen modified atmosphere. The selected strains were the most abundant ones on oxygen-packaged meat as determined in our previous studies. *C. divergens* TMW2.1577 and *C. maltaromaticum* TMW2.1581 were isolated from skinless chicken breast by Höll et al. (2016), *L. gelidum* subsp. *gelidum* TMW2.1618, and *L. gelidum* subsp. *gasicomitatum* TMW2.1619 from beef steaks by Hilgarth et al. (2018) and *B. thermosphacta* TMW2.2101 from minced beef (Hilgarth et al., 2019).

### Genome Sequencing

Genomic DNA of *L. gelidum* subsp. *gelidum* TMW2.1618, *L. gelidum* subsp. *gasicomitatum* TMW2.1619, *C. divergens* TMW2.1577, and *B. thermosphacta* TMW2.2101 was extracted using the E.Z.N.A. Bacterial DNA Kit from Omega (Norcross, GA, United States). Genomes of *L. gelidum* subsp. *gelidum* and *L. gelidum* subsp. *gasicomitatum* were sequenced using SMRT by GATC Biotech (Konstanz, Germany) with a PacBio RSII sequencer. Genomes of *C. divergens* and *B. thermosphacta* were sent to the microbiome core facility of the Institute of Food & Health (TUM Freising, Germany) for Illumina MiSeq shotgun sequencing. Genome accession numbers are CP017196 (*L. gelidum* subsp. *gelidum* TMW2.1618), CP017197 (*L. gelidum* subsp. *gasicomitatum* TMW2.1619), RSDV000000001 (*C. divergens* TMW2.1577), CP016844 (*C. maltaromaticum* TMW2.1581), and RSDU000000001 (*B. thermosphacta* TMW2.2101).

### Bacterial Culture Conditions and Meat Simulation Medium

Brain heart infusion (BHI) medium (Roth, Karlsruhe, Germany) was used for preparing pre-cultures. After overnight cultivation at 25°C, cells were stored at −80°C as cryopreserved stock cultures in glycerol enabling to use aliquots from the same stock culture for all incubation experiments.

In order to create a sterile simulation system *in vitro* without interfering background microbiota, we established an experimental setup, which reflects conditions of meat packaged under modified atmosphere. Therefore, we created a meat simulation media (MSM), which contains main components of real meat. The basic medium consisted of meat extract (Merck, Darmstadt, Germany). Cell growth of each species was determined under different meat extract concentrations. The lowest concentration exhibiting measureable cell growth was chosen for each species and used in the experiment (*C. diverg*ens 12.5 g l^–1^, *B. thermosphacta* 50 g l^–1^, and *C. maltaromaticum*, *L. gelidum* subsp. *gelidum* and *Leuconostoc gelidum* subsp. *gasicomitatum* 100 g l^–1^). As meat extract is free of fat, but fat represents one major substrate component on fresh meats, 0.05 mM Tween80 (Gerbu Biotechnik GmbH, Heidelberg, Germany) and 0.5% glycerol (Gerbu Biotechnik GmbH, Heidelberg, Germany) were added to the medium. Tween80 consists of polyethoxylated sorbitan and oleic acid. Oleic acid is the most abundant fatty acid found in animal fats and is often used as an additive in media to provide bacteria with oleic acid (Man et al., 2008; Reitermayer et al., 2018). In a subsequent experiment, influence of heme on bacterial growth was tested by addition of 2 μg/ml heminchloride (Roth, Karlsruhe, Germany) solved in dimethylsulfoxide (99.8%) (Roth, Karlsruhe, Germany) after autoclaving due to its light and heat instability. For optimal growth of both *Leuconostoc* subspecies and *B. thermosphacta*, 0.5% glucose monophosphate (Merck, Darmstadt, Germany) was added. Finally, pH of the prepared MSM was adjusted to 5.8 with 100% lactic acid reflecting the approx. pH of meat. MSM was used for all experiments.

### Experimental Setup

The main experiment was carried out in 1 liter (1 l) high pressure glass bottles (Schott, Mainz, Germany) filled with 0.5 l MSM. Bottles were locked by a gas tight butyl rubber. Two sampling cannula permitted measurement of headspace gas composition and cell growth during cultivation. The headspace gas composition was measured by a PA 7.0 gas analyzer (Witt-Gasetechnik, Witten, Germany). A Fibox 3 LCD trace oxygen meter and autoclavable SP-PSt3 oxygen sensor spots (PreSens, Regensburg, Germany) were used to monitor oxygen saturation values of the media. The experimental setup is shown in Supplementary Figure S1.

Before inoculation, the MSM was adapted to room temperature and degassed with a specific gas mixture (70% O_2_/30% CO_2_) representing the headspace gas composition of high-oxygen packaged meat. Gassing was performed until constant starting values of 70% oxygen saturation of the medium and a headspace gas composition of 70% oxygen and ∼25% carbon dioxide was achieved. Afterward, gas flow was stopped and bottles were locked gas tight. Thus, the fermentation medium MSM mimicking meat and the headspace of the fermentation bottles the headspace of high oxygen MAP meat packages.

Media were inoculated with a bacterial suspension (OD_590_ = 0.1) and stirred at 160 rpm. Oxygen saturation, temperature, pH-value, and total viable count as colony forming units (CFUs) of the medium, as well as the gas composition and pressure of the headspace were monitored over 60 h for all selected bacteria. Whole experiments were performed in triplicates.

### Calculation of the Oxygen Uptake Rate per Cell

First, dissolved oxygen (DO) of the medium was calculated based on the percentage of oxygen saturation, temperature, and pressure of the medium. Therefore, we used the oxygen unit calculator provided by PreSens (Regensburg, Germany). Based on the DO we calculated the oxygen uptake rate (OUR) given as

(1) OUR=Ka*L([O2]-*[O2])-d[O2]/dt

with [O_2_] representing the measured DO concentration and [O_2_]^∗^ the DO concentration of oxygen saturated medium. In order to incorporate the oxygen transfer rate from atmosphere to liquid interface in the calculation, the volumetric mass-transfer coefficient *k_*L*_a* was calculated by the gassing-out method previously used by Gupta and Rao (2003). For this, 1 liter (1 l) high pressure glass bottles (Schott, Mainz, Germany) were filled with 500 ml of the respective media. Nitrogen was bubbled into the liquid until reaching anoxia. After oxygen expulsion, headspace was replaced with 70% oxygen and ∼25% carbon dioxide. Finally, the stirrer was turned on, and the oxygen entry into the respective media was measured over time. The *k_*L*_a* was calculated by the following equation:

(2) $$-k_{L}\,a=\Delta \,\ln\left({\left[{\text{O}}_{2}\right]}^{*}-\left[{\text{O}}_{2}\right]\right)/\Delta \,t.$$

In order to compare the oxygen consumption rate between different species, the different cell counts had to be considered for the oxygen uptake. A mathematic model to represent dynamic cell growth over time was developed in order to enable calculation of OUR per cell. Measured CFU values over time were fitted by a sigmoidal cell growth curve based on Boltzmann function with the software OriginPro (Version b9.5.0.193, Northampton, MA, United States). Based on the resulting equation *f*(*x*) we calculated the antiderivative *F*(*x*), which was then used to determine the integral between two sampling time points *t*_1_ and *t*_2_. The general equation is given as

(3) $$\int_{t_{1}}^{t_{2}}\left(A_{2}+\frac{A_{1}-A_{2}}{1+e^{x-x_{0}/\text{d}\,x}}\right)\,\text{d}\,x$$

with *A*_2_, *A*_1_, *x*, *x*_0_, and d*x* corresponding to specified parameters of the Boltzmann function. The integral of two sampling time points was then divided by 2 and the $\left(\int_{t_{1}}^{t_{2}}\text{d}\,x\right)/2$ was added to the integral of $t_{1}\,\int_{0}^{t_{1}}$. The resulting integral (*y*) was then used to calculate the true CFU development between the two sampling time points. Therefore, the Newton–Raphson method was applied to calculate *x* of the following general equation:

(4) $$y=\left(\frac{A^{*}\,\ln\left(e\,\frac{B}{D}-\frac{C^{*}\,x}{E}+1\right)}{F}+E^{*}\,x\right)$$

*A*–*F* are constants of the equation. The calculated *x*-value was inserted into the Boltzmann function to calculate the fitted CFU value of the time point *t*_2_. This calculated CFU values were finally used to calculate the OUR per cell. OUR and DO per cell were calculated separately for each replicate of each species. Afterward, average values of the three independent replicates were calculated and subjected to statistical analysis (see section “Statistic Data Analyzation”).

### Total Oxygen Consumption Balance of the Headspace

In order to determine the total oxygen consumption quantity of the headspace atmosphere over 60 h, we first calculated the partial pressure of oxygen in the headspace as followed:

(5) $$p_{{\text{O}}_{2}}=p_{\text{total}}^{*}\,x_{{\text{O}}_{2}}.$$

*x*_*O_2*_ was defined as the percentage of oxygen measured by the gas analyzer. By applying the ideal gas equation given as

(6) $$p^{*}\,V=n^{*}\,R^{*}\,T$$

We calculated the amount of oxygen in the headspace atmosphere. As oxygen consumption is depending on the dynamic change of cells over 60 h, the total amount of oxygen consumed was divided by the total integral of the CFU curve given as

(7) $$\int_{t_{\text{start}}}^{t_{\text{end}}}\left(A_{2}+\frac{A_{1}-A_{2}}{1+e^{x-x_{0}/\text{d}\,x}}\right)\,\text{d}\,x.$$

The total oxygen consumption in 60 h and per single cell was calculated for each replicate of each species. Afterward, average values of the three independent replicates were calculated and subjected to statistical analysis (see section “Statistic Data Analyzation”).

### Cell Growth With Heminchloride, Menaquinone-4, and Hydrogen Peroxide

Bacteria were cultivated in MSM medium (OD_600_ = 0.1), supplemented with either heminchloride, menaquinone-4 (Sigma-Aldrich, St. Louis, MO, United States), or both, as described by Brooijmans et al. (2009). An oxidative stress test was performed in MSM medium supplemented with different concentrations of hydrogen peroxide (0.5–0.008%) (Merck, Darmstadt, Germany). Cell growth was monitored in 96-well plates (Sarstedt, Nümbrecht, Germany) with a FLUOstar Omega microplate reader (BMG Labtech, Ortenberg, Germany) over 48 h at 600 nm. Minimal inhibitory concentration (MIC) of hydrogen peroxide was determined for each species. Experiments were performed in technical triplicates. All data shown represent average values.

### Statistic Data Analyzation

Differences between species regarding OUR or total oxygen consumption were tested for significance using the open source software RStudio ver. 3.3.0 (RStudio Inc., Boston, United States). First, data were tested for normal distribution (*P* > 0.05) and homology of variance (*P* < 0.05) by Shapiro–Wilk normality test and Bartlett test followed by a one-way analysis of variance (ANOVA). At last, significant differences (*P* < 0.05) between means were calculated by *post hoc* Tukey test.

## Results

### Cell Growth Under High Oxygen Atmosphere and pH Change

All bacteria were able to grow under an atmosphere containing 70% oxygen and 30% carbon dioxide. Nevertheless, differences between the five spoilers could be seen. High lag phases were observed for *C. divergens* TMW2.1577 and *B. thermosphacta* TMW2.2101 (33 h, 22 h) compared to *C. maltaromaticum* TMW2.1581 (16 h) and the strains of both *Leuconostoc* species (12 h). These differences were classified as significant between the species. *C. divergens* TMW2.1577 exhibited a strong decrease of the viable cell count prior to the exponential phase. Furthermore, a strong decrease of the CFU was observed for *B. thermosphacta* TMW2.2101 after 25 h of fermentation (Supplementary Figure S2). During cultivation, pH evolution was recorded for all species. All bacteria acidified the fermentation media with *C. maltaromaticum* TMW2.1581 exhibiting less acidification (final pH 4.9) than the other species (final pH 4.6). pH values started decreasing as soon as bacterial cell growth was detectable (Supplementary Figure S3).

### Oxygen Consuming Capacity of LAB and *B. thermosphacta*

**FIGURE 1:**
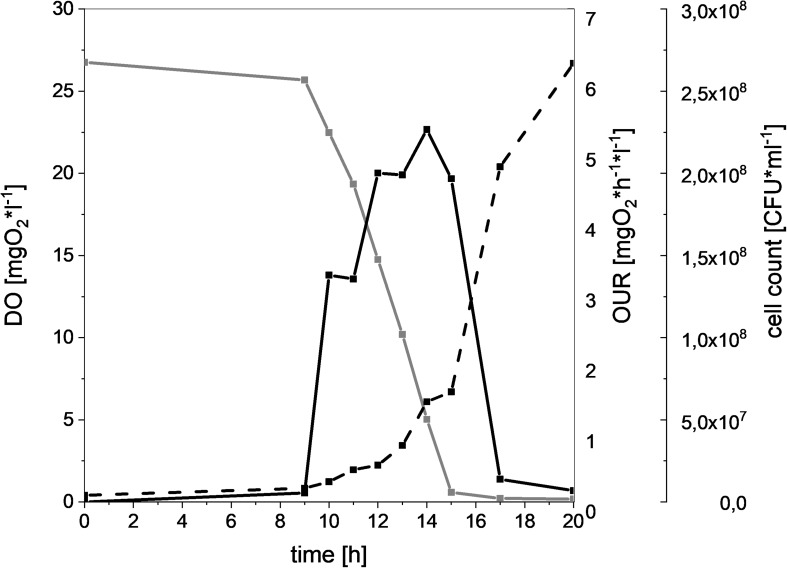
Oxygen consumption and cell growth of *L. gelidum* subsp. *gelidum* under high oxygen atmosphere. Dissolved oxygen [mgO_2_^∗^l^–1^] (gray solid line), oxygen uptake rate [mgO_2_^∗^h^–1*^l^–1^] (black solid line), and cell count [CFU^∗^ml^–1^] (black dotted line) of *L. gelidum* subsp. *gelidum* TMW2.1618 replicate 1.

In order to compare the oxygen consuming capacity of different meat-spoiling bacteria, we monitored the dynamic cell growth (CFU), oxygen consumption (DO), and OUR in a MSM. Results for *L. gelidum* subsp. *gelidum* TMW2.1618 can be seen in Figure 1 as a typical example. There was a clear correlation of increasing CFU and oxygen uptake for all bacteria, as the amount of DO starts decreasing once an increase of the CFU was detectable. Additionally, OUR increased proportional to oxygen consumption. Oxygen depletion within the medium occurred within 15 h, resulting in a rapid OUR decrease, as the diffusion of oxygen from the headspace was comparably low. These correlations could be seen for all spoilers (Supplementary Figure S4).

**FIGURE 2:**
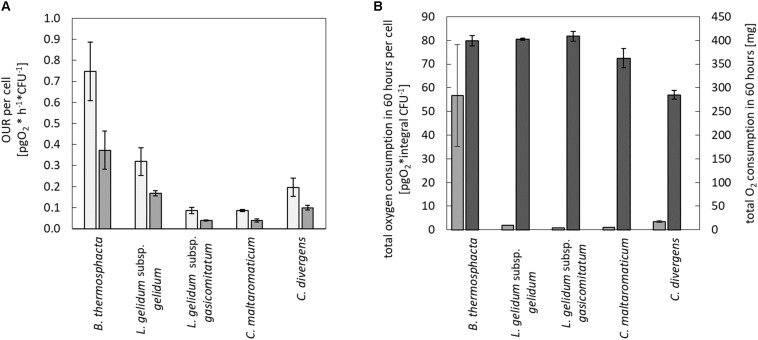
Quantification of the oxygen consumption in the medium and headspace for all species. Oxygen consumption was monitored for all species in the media and in the headspace. Values represent average values of three independent replicates. In the medium, **(A)** maximum (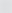) and average (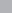) oxygen uptake rate was calculated until oxygen limitation. In the headspace, **(B)** total oxygen consumption in 60 h was calculated for all cells (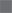) and for a single cell (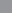).

Calculated values of the maximum and the average OUR per cell are shown in Figure 2A. In direct comparison to the four tested LAB species, *B. thermosphacta* TMW2.2101 showed a significantly higher maximum OUR per cell. *B. thermosphacta* was able to consume up to 0.748 pg of oxygen per hour per cell, whereas other spoilers consumed about 70% less (*C. divergens*, *C. maltaromaticum*, and *L. gelidum* subsp. *gasicomitatum*). In detail, *B. thermosphacta* TMW2.2101 had a twofold higher maximum OUR compared to *L. gelidum* subsp. *gelidum* TMW2.1618, fourfold higher maximum OUR compared to *C. divergens* TMW2.1577, and eightfold higher maximum OUR compared to *L. gelidum* subsp. *gasicomitatum* TMW2.1619 and *C. maltaromaticum* TMW2.1581. The differences were classified as significant for *L. gelidum* subsp. *gelidum* (*P* = 0.012), highly significant for *C. divergens* (*P* = 0.002), and extremely significant for *C. maltaromaticum* and *L. gelidum* subsp. *gasicomitatum* (*P* = 0.0005; *P* = 0.0005), respectively. Within the strains of the LAB species, *L. gelidum* subsp. *gelidum* TMW2.1618 exhibited a significant higher maximum OUR per cell compared to *L. gelidum* subsp. *gasicomitatum* TMW2.1619 (*P* = 0.015) and *C. maltaromaticum* TMW2.1581 (*P* = 0.015). Comparing the average oxygen consumption per cell, *B. thermosphacta* TMW2.2101 again showed highest OUR compared to the other species with *P*-values between 0.02 and 0.002 representing significant and highly significant differences. Within the LAB species, *L. gelidum* subsp. *gelidum* TMW2.21618 also exhibited significant differences to all other species. Furthermore, the average OUR of *C. divergens* TMW2.1577 was significantly higher than the average OUR of *L. gelidum* subsp. *gasicomitatum* TMW2.1619 and *C. maltaromaticum* TMW2.1581 (*P* = 0.005; *P* = 0.005).

### Changes of the Headspace Atmosphere and Total Oxygen Consumption

**FIGURE 3:**
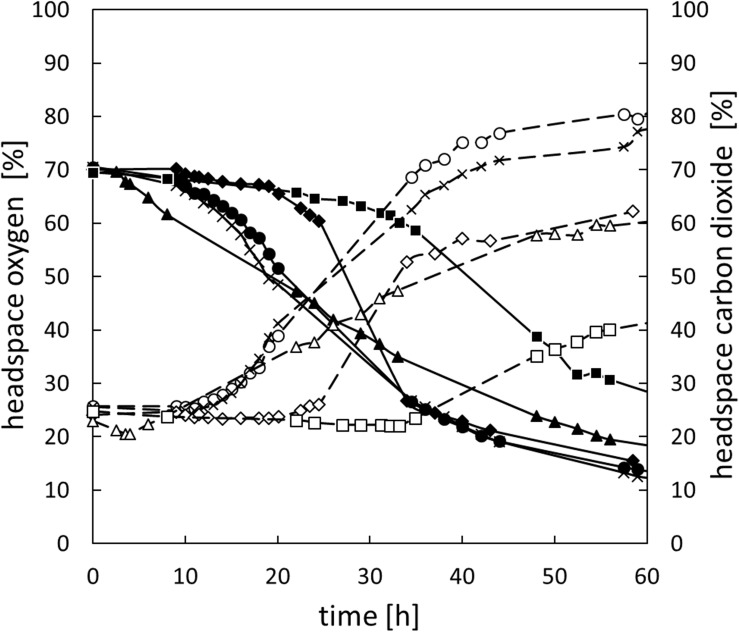
Change of the headspace gas composition for all species over 60 h. *B. thermosphacta* TMW2.2101 (◆), *C. divergens* TMW2.1577 (■), *C. maltaromaticum* TMW2.1581 (▲), *L. gelidum* subsp. *gelidum* TMW2.1618 (⚫), *L. gelidum* subsp. *gasicomitatum* TMW2.1619 (x). Solid line, black symbol: oxygen concentration. Dotted line, white symbol: carbon dioxide concentration. Values represent average values of three independent replicates.

In addition to changes of the oxygen concentration within the medium, changes in the headspace atmosphere of the bottles could also be detected (Figure 3). Starting from 70% oxygen in the headspace, values decreased for all bacteria to <30% in 60 h. The total oxygen consumption per cell during the whole fermentation period was calculated (Figure 2B). All bacteria consumed similar total amounts of oxygen during 60 h of fermentation. Nevertheless, *B. thermosphacta* TMW2.2101 exhibited highest total oxygen consumption with respect to the total CFU. One cell of *B. thermosphacta* TMW2.2101 was able to consume 31 times more oxygen compared to the other species over the whole incubation period. Total oxygen consumption of *B. thermosphacta* was significantly different compared to all four LAB species, with *P*-values of <0.019.

Carbon dioxide levels were reciprocally changing to the oxygen concentration, i.e., rising CO_2_ levels with decreasing oxygen concentrations starting from initial values of 25% carbon dioxide in the headspace up to 40% for *C. divergens* TMW2.1577 and >60% for the other species after 60 h of cultivation (Figure 3). Highest carbon dioxide production per cell was detected for *B. thermosphacta* TMW2.2101. One cell of *B. thermosphacta* TMW2.2101 was able to produce 34 times more carbon dioxide compared to the other species. Overall, *C. divergens* TMW2.1577 inoculated bottles showed the weakest change in the headspace atmosphere compared to the other species, despite the medium of *C. divergens* exhibited the highest diffusion coefficient (12.5 g/l *K_*L*_A* = 0.0653; 50 g/l *K_*L*_A* = 0.0172; 100 g/l *K_*L*_A* = 0.0054) (Supplementary Figure S5).

### Respiration With Heme and Menaquinone and Resistance to Oxidative Stress

Furthermore, all species were tested for potential heme-based respiration by heme and menaquinone addition (Table 1). No significant enhanced cell growth was observed by the addition of menaquinone for any species. Contrary, all species exhibited significantly higher cell growth by the addition of heme (*P*-values of 0.01–2.4 ^∗^ 10^–6^). Addition of heme and menaquinone was not significantly different to the cell growth with only heme. Susceptibility of meat-spoiling bacteria to oxidative stress was tested by addition of hydrogen peroxide (Table 2). The minimal inhibitory concentration of hydrogen peroxide for each species was determined in technical triplicates. All replicates showed the same MIC value. *B. thermosphacta* TMW2.2101 exhibited a four to seven times higher tolerance to oxidative stress compared to all tested LAB species.

**TABLE 1:** Heminchlorid (heme) or menaquinone (MQ) enhanced cell growth (OD_600_) of *B. thermosphacta* TMW2.2101, *C. divergens* TMW2.1577, *C. maltaromaticum* TMW2.1581, *L. gelidum* subsp. *gelidum* TMW2.1618, *L. gelidum* subsp. *gasicomitatum* TMW2.1619.

	**Heme**		**MQ**		**Heme and MQ**	
	**+**	**−**	**+**	**−**	**+**	**−**
*B. thermosphacta* TMW2.2101	1.364	0.990	0.992	1.014	1.316	0.980
*L. gelidum* subsp. *gelidum* TMW2.1618	1.250	1.031	1.088	1.024	1.297	1.213
*L. gelidum* subsp. *gasicomitatum* TMW2.1619	1.412	0.872	0.950	0.936	1.411	0.902
*C. maltaromaticum* TMW2.1581	1.845	1.317	1.399	1.376	1.818	1.308
*C. divergens* TMW2.1577	0.855	0.610	0.634	0.615	0.861	0.627

**TABLE 2:** Minimal inhibitory concentration (MIC) values of the species *B. thermosphacta* TMW2.2101, *C. divergens* TMW2.1577, *C. maltaromaticum* TMW2.1581, *L. gelidum* subsp. *gelidum* TMW2.1618, *L. gelidum* subsp. *gasicomitatum* TMW2.1619 to hydrogen peroxide.

	**MIC (%)**
*B. thermosphacta* TMW2.2101	0.090
*L. gelidum* subsp. *gelidum* TMW2.1618	0.016
*L. gelidum* subsp. *gasicomitatum* TMW2.1619	0.020
*C. maltaromaticum* TMW2.1581	0.024
*C. divergens* TMW2.1577	0.012

### Genomic Prediction of a Respiratory Chain, Citric Acid Cycle, and Tolerance to Oxidative Stress

In order to explain the different OURs determined in this study, a genomic analysis of all species was performed with respect to presence or absence of relevant genes (Table 3). We focused on genes encoding enzymes, which catalyze single oxygen consuming reactions, e.g., the pyruvate oxidase or glycerol-3-phosphate oxidase and on genes encoding for proteins needed to establish a minimum functional respiratory chain. This comprises a NADH dehydrogenase, a menaquinone, terminal cytochrome oxidase, a F_0_F_1_-ATPase, and genes needed for either heme biosynthesis or uptake. *C. divergens* TMW2.1577 and *C. maltaromaticum* TMW2.1581 possess all genes for a functional respiratory chain including a heme uptake system, whereas both *Leuconostoc* subspecies lack genes needed for heme uptake as well as biosynthesis. *B. thermosphacta* TMW2.2101 also possesses all genes needed to establish a functional respiratory chain, biosynthesize heme and form an additional cytochrome aa_3_ and ba_3_ terminal oxidase. Regarding other single oxygen consuming reactions, all bacteria possess genes for a glycerol-3-phosphate oxidase, a NADH oxidase, and an acetolactate synthase. Genes, encoding a pyruvate oxidase could only be found for *B. thermosphacta* TMW2.2101 and *L. gelidum* subsp. *gasicomitatum* TMW2.1619. Furthermore, both *Leuconostoc* species encode for a pyridoxine 5′-phosphate oxidase, which could not be found in strains of the other species. Regarding mechanisms to cope with oxidative stress, *B. thermosphacta* TMW2.2101 contains H_2_O_2_-producing superoxide dismutase (SOD) as well as catalase and multiple peroxidases to reduce H_2_O_2_. *Leuconostoc* species do not possess catalase and SOD, but only encode for a peroxiredoxin and two peroxidases. *C. divergens* and *maltaromaticum* possess SOD, a catalase, and a glutathione peroxidase. Additionally, *C. divergens* TMW2.1577 possesses a peroxiredoxin and *C. maltaromaticum* TMW2.1581 an additional peroxidase. With respect to a citric acid cycle, *B. thermosphacta* TMW2.2101 does possess genes encoding a citrate synthase, aconitate hydratase, isocitrate dehydrogenase, fumarate hydratase, and isocitrate lyase whereas *L. gelidum* subsp. *gasicomitatum* TMW2.1619 only encodes for a isocitrate lyase. None enzyme of the citric acid cycle could be found for *L. gelidum* subsp. *gelidum* TMW2.1618. Both *Carnobacteria* species encode for a citrate synthase, aconitate hydratase, and isocitrate dehydrogenase. *C. maltaromaticum* TMW2.1581 also encodes for a fumarate hydratase. Thus, none of the five species possesses all genes to establish a functional citric acid cycle (Supplementary Table S1).

**TABLE 3:** List of genes needed to establish an aerobe respiratory chain as well as single oxygen consuming reactions for *B. thermosphacta* TMW2.2101, *L. gelidum* subsp. *gelidum* TMW2.1618, *L. gelidum* subsp. *gasicomitatum* TMW2.1619, *C. maltaromaticum* TMW2.1581, and *C. divergens* TMW2.1577.

	***B. thermosphacta* TMW2.2101**	***L. gelidum* subsp. *gelidum* TMW2.1618**	***L. gelidum* subsp. *gasicomitatum* TMW2.1619**	***C. divergens* TMW2.1577**	***C. maltaromaticum* TMW2.1581**
**Biosample**	**EHX26**	**BHS02**	**BHS03**	**EH150**	**BFC23**
**NADH dehydrogenase**					
NADH dehydrogenase (*ndh*)	06170	01115	01095	06635	01885
**Menaquinone biosynthesis**					
1,4-Dihydroxy-2-naphthoate prenyltransferase (*men*A)	11995	06815	07110	09580^∗^	01875^∗^
1,4-Dihydroxy-2-naphthoyl-CoA synthase (*men*B)	11975	00095	00095	10860	12070
*o*-Succinylbenzoate synthase (*men*C)	08125	04505	04575	11290	11525
2-Succinyl-5-enolpyruvyl-6-hydroxy-3- cyclohexene-1-carboxylic-acid synthase (*men*D)	11985	04515	04585	11300	11535
*o*-Succinylbenzoate–CoA ligase (*men*E)	11970	00265	00210	10855	12065
Isochorismate synthase (*men*F)	11990	04520	04590	11305	11540
2-Succinyl-6-hydroxy-2, 4-cyclohexadiene-1-carboxylate synthase (*men*H)	11980	04510	04580	11295	11530
2-Methoxy-6-polyprenyl-1,4-benzoquinol methylase (*ubi*E)	08745	00270	00215		00240^∗^
**Cytochrome aa3 quinol oxidase**					
Cytochrome aa3 quinol oxidase subunit I	07625				
Cytochrome aa3 quinol oxidase subunit II	07620				
Cytochrome aa3 quinol oxidase subunit III	07630				
Cytochrome aa3 quinol oxidase subunit IV	07635				
**Cytochrome bd ubiquinol oxidase**					
Cytochrome ubiquinol oxidase subunit I (*cyd*A)	07685	02640	02605	06670	01825
Cytochrome d ubiquinol oxidase subunit II (*cyd*B)	07690	02645	02610	06665	01830
Thiol reductant ABC exporter subunit (*cyd*C)	07700	02655	02620	06655	01835
Thiol reductant ABC exporter subunit (*cyd*D)	07695	02650	02615	06660	01840
**Cytochrome O ubiquinol oxidase**					
Cytochrome O ubiquinol oxidase		05340	05410		00350
**F_0_F_1_-ATP synthase**					
F0F1 ATP synthase subunit A	08060	00690	00670	05855	01335
ATP synthase F0 subunit B	08070	00700	00680	05845	01345
F0F1 ATP synthase subunit C	08065	00695	00675	05850	01340
F0F1 ATP synthase subunit alpha	08080	00710	00690	05835	01355
F0F1 ATP synthase subunit beta	08090	00720	00700	05825	01365
F0F1 ATP synthase subunit gamma	08085	00715	00695	05830	01360
F0F1 ATP synthase subunit delta	08075	00705	00685	05840	01350
F0F1 ATP synthase subunit epsilon	08095	00725	00705	05820	01370
**Heme biosynthesis**					
Glutamyl−tRNA reductase (*hem*A)	04720			01100	07045
Porphobilinogen synthase (*hem*B)	04730				
Hydroxymethylbilane synthase (*hem*C)	04725				
Uroporphyrinogen-III synthase (*hem*D)	04735				
Uroporphyrinogen decarboxylase (*hem*E)	04715				
Coproporphyrinogen III oxidase (oxygen independent) (*hem*N)	11840			01505	06610
Protoporphyrinogen IX dehydrogenase (*hem*G)	03920			00585	08360
Ferrochelatase (*hem*H)	03925			07970	01065
Protoheme IX farnesyltransferase	01275				
Heme A synthase	01280				
**Heme uptake and homeostasis**					
Sortase B protein-sorting domain-containing protein (*isd*A) Hypothetical protein	11590^∗^			01365^∗^	11300^∗^
Iron surface determinant B (*isd*B)					
Iron surface determinant H (*isd*H)					
Iron surface determinant C (*isd*C)	11585			01360	11305
Iron surface determinant E (*isd*E)	11595			01370	11295
Heme ABC transporter ATP-binding protein (*isd*D)		00405	00375	01380^∗^	07565 10175 15265
Iron ABC transporter permease (*isd*F homolog)	11600			01375^∗^	11290
Iron surface determinant G (*isd*G)	10855			12435	13075
**Single oxygen consuming reactions**					
Pyruvate oxidase	03750		04365		
Glycerol-3-phosphate oxidase/dehydrogenase	08435	05735	05720	04895	09570
NADH oxidase	09890	04450	04520	00705	08530 11520 10095
Acetolactate synthase (*Als*S) (alpha acetolactate -> 2.3-butanedione)	01770	06300	06360	03770	03635
Pyridoxine 5′-phosphate oxidase		04395	04470		
**Antioxidants**					
Catalase	11130			10970	11875
Superoxide dismutase (*SOD*)	06805			04005	07340
2-*Cys* peroxiredoxin		07480	07730		03975
Glutathione peroxidase	03205	04205	04195	06705	12965
Other peroxidases	03930 10115	01155	01135	03590	

*^∗^Putative gene function only. All predicted gene functions are based on NCBI genome annotation (PGAP) and have been further confirmed by manual BLAST search. Numbers correspond to the gene locus tag of the genome assembly.*

## Discussion

Recent monitoring studies on high-oxygen MAP beef, minced beef, and poultry have demonstrated a significant decrease of the oxygen content in the headspace atmosphere upon storage (Höll et al., 2016; Hilgarth et al., 2018, 2019). Upon oxygen consumption, the microbiome composition and general metabolism switches to amino acid conversion causing putrid spoilage of the product and marked by respective sensorial changes. Consequently, fast oxygen consumption should accelerate the atmosphere-triggered microbiome switch and affect sensorial shelf life. In our study, we found marked differences in oxygen consumption within most abundant strains of relevant meat spoiling species. As a result, initial contamination with specific CO_2_-tolerant oxygen consumers should enable forecast of the onset of meat spoilage in high oxygen MAP.

### Cell Growth of Bacteria With 70% Oxygen/30% Carbon Dioxide Atmosphere

In this study, both *Leuconostoc* species showed comparable low lag phases and high cell growth, indicating less sensitivity to high oxygen and carbon dioxide concentrations. Contrary, *C. divergens* TMW2.1577 exhibited a long lag-phase with a strong decrease of the viable cell count prior to the exponential phase indicating a necessary adaptation of the cells to modified atmosphere. *B. thermosphacta* TMW2.2101 exhibited fast generation times while the medium was aerobic, but was highly sensitive to oxygen limitation, resulting in a drop of cell counts at the time point of oxygen depletion (25 h). This is in accordance to previous assertiveness studies on minced beef and poultry done by Höll et al. (2016) and Hilgarth et al. (2019), which revealed a decrease of the relative abundance of *B. thermosphacta* as soon as oxygen in the package was limited or depleted, indicating that only a subpopulation is able to adjust to anoxic conditions. Furthermore, the pH drop after 25 h of fermentation might also contribute to the decrease of the CFU of *B. thermosphacta* TMW2.2101.

### Oxygen Consumption by Different LAB and *B. thermosphacta*

All bacteria analyzed in this study were able to consume oxygen in the meat-derived model system and were concomitantly producing CO_2_. The amount of produced CO_2_ was not always directly proportional for each species, indicating that CO_2_ might have also been produced heterofermentatively. Despite the high concentration of CO_2_, all bacteria studied in this work will likely contribute to oxygen consumption also in meat packages. Nevertheless, *B. thermosphacta* TMW2.2101 exhibited significantly higher OURs compared to the strains of the other four LAB species. A decrease of DO by other strains of *B. thermosphacta* and *C. divergens* as well as *L. mesenteroides* have also been proven by Borch and Molin (1989). Taking together, these results prove that *B. thermosphacta* TMW2.2101 consumes available oxygen more rapidly than LAB. Thus, *B. thermosphacta* appears to have a higher contribution to the oxygen decrement in meat packages *in situ* compared to LAB at similar CFU. The similar amounts of total oxygen consumed (indicated by a similar headspace composition change) after 60 h of fermentation for all species can be explained by a higher total cell count of the LAB at the time when the media became anoxic compared to *B. thermosphacta*, in which only a subpopulation was able to cope with oxygen limitation, but exhibited high oxygen consumption rates.

### Genomic Prediction of Metabolic Pathways Involved in Oxygen Consumption

In order to explain the high OUR of *B. thermosphacta* TMW2.2101 compared to the LAB species as well as its sensitivity to oxygen limitation, we investigated their genomic settings to form a functional aerobic respiratory chain, other oxygen consuming reactions as well as mechanisms to cope with oxidative stress. In a previous review done by Lechardeur et al. (2011), a minimum respiration machinery has been described for specific LAB. This respiratory machinery consists of an electron donor (NADH dehydrogenase), an electron shuttle (menaquinone), a terminal electron acceptor (cytochrome quinol oxidase), and a proton gradient-driven F_0_F_1_-ATP synthase. Additionally, heme is required as an important cofactor for cytochrome oxidase activity. Heme can either be biosynthesized by bacteria themselves or be untaken from extracellular sources. Furthermore, as true respiration is always coupled to the citric acid cycle, we analyzed all species for their potential to perform this metabolic pathway. According to the genomes, all analyzed LAB strains and *B. thermosphacta* TMW2.2101 are predicted as unable to form a functional citric acid cycle. However, formation of a respiratory chain – even without a citric acid cycle – allows effective coupling of fermentation with respiration through interconversion of NADH/NAD^+^, i.e., fermentation-borne NADH can be regenerated to NAD+ via respiration and *vice versa*, resulting in a competitive advantage by higher energy yield.

*Leuconostoc gelidum* subsp. *gelidum* TMW2.1618 and *L. gelidum* subsp. *gasicomitatum* TMW2.1619 encode for all genes needed to build up a minimal respiratory chain except some genes needed for the heme uptake system. The enhanced cell growth measured for *L. gelidum* subsp. *gasicomitatum* TMW2.1619 by the addition of heme indicates that this strain is be able to build up a functional respiratory chain. This is in concordance to previous studies conducted by Johansson et al. (2011) and Jääskeläinen et al. (2013). They were able to show a heme-based respiration of different strains of *L. gelidum* subsp. *gasicomitatum in vitro* and *in situ*. No growth promoting effect of heme was yet described for *L. gelidum* subsp. *gelidum* strains (Rahkila et al., 2014). However, our study also revealed a significant increase of cell growth of *L. gelidum* subsp. *gelidum* TMW2.1618 by addition of heme indicating that this strain is able to respire. In addition, both *Leuconostoc* subspecies also encode for other oxygen consuming enzymes which could contribute to the oxygen consumption.

Both *Carnobacterium* species possess the genetic setting to build a respiratory chain. Regarding electron transport shuttles, we did not find the gene for the last step of the menaquinone biosynthesis pathway (*ubiE*) for the strain *C. divergens* TMW2.1577. Nevertheless, menaquinone supplementation to the medium did not promote cell growth of both species in our study, suggesting that both are able to synthesize menaquinone themselves. Addition of heme to the media resulted in enhanced cell growth for both *Carnobacterium* strains indicating uptake of extracellular heme. Indeed, we found a putative *isdA* gene, encoded as sortase depending protein (SDP), which is thought to bind free heme or accept heme from the hemoglobin receptor *isdH* (Iskandar et al., 2017). Thus, both species are predictively able to uptake not only free heme, but also accept heme bound in the protein complex hemoglobin on meats. Taking together, these findings suggest that the strains of both species are able to respire by aerobe respiratory chain activity. Similar has previously been shown in other studies (Brooijmans et al., 2009; Lechardeur et al., 2011). Nevertheless, genomes of both species also encode for other single oxygen consuming enzymes, which might also contribute to oxygen consumption in meat packages.

Compared to LAB, *B. thermosphacta* TMW2.2101 also possesses genes encoding heme A, B, and O biosynthesis. Additionally, it possesses genes encoding proteins to establish a heme-uptake system. When offered free heme, *B. thermosphacta* TMW2.2101 showed enhanced cell growth, indicating uptake of extracellular heme instead of biosynthesis to safe energy or does both, resulting in a higher amount of heme for respiration. We were also able to detect all three subunits of a cytochrome a_3_ quinol oxidase as well as an additional a-type cytochrome oxidase. Thus, *B. thermosphacta* TMW2.2101 can predictively build up two additional cytochromes, aa_3_ and ba_3_, compared to the other LAB. A similar cytochrome composition of *B. thermosphacta* has previously been described by Gil et al. (1992). As shown in this study, the abundance of different cytochromes in *B. thermosphacta* is dependent on extracellular oxygen concentrations. Thus, the high OUR of *B. thermosphacta* TMW2.2101 could be explained by varying cytochrome composition and possible adaptation to varying oxygen concentrations. Nevertheless, *B. thermosphacta* TMW2.2101 also encodes for other additional enzymes which can contribute to oxygen consumption.

We also analyzed resistance of all species to respiration-borne reactive oxygen species. Previous studies regarding oxidative stress showed that superoxide radicals can arise from aerobe respiratory activity. During respiration, 0.1–2% of the molecular oxygen is incompletely reduced, resulting in the formation of superoxide radicals (Madamanchi and Runge, 2007; Li et al., 2013). These superoxide radicals can be converted to hydrogen peroxide by the SOD (Turrens et al., 1985), but have to be further detoxified. *B. thermosphacta* TMW2.2101 exhibited highest tolerance to hydrogen peroxide compared to the LAB enabling a higher respiratory activity by the presence of more antioxidant enzymes within its genome.

## Conclusion

This study is the first one quantifying oxygen consumption of meat spoiling bacteria in a meat simulation media. While conditions may differ *in situ* to some degree, the media enables to study the behavior of the single strains in a defined, sterile environment. Furthermore, we explained the mechanistic differences with comparative genomic and physiologic analyses. *B. thermosphacta* TMW2.2101 exhibited the highest oxygen consumption activity due to its high OUR. This can be explained by its cytochrome composition and oxidative stress tolerance. The initial contamination level of *B. thermosphacta* can therefore serve as a predictive indicator for the onset of spoilage in high oxygen MAP meat.

## Data Availability Statement

The datasets for this study can be found in the NCBI repository; CP017196, CP017197, RSDU00000000.1/, RSDV00000000.1/, and CP016844.

## Author Contributions

SK designed the study, performed the experiments and data evaluation, and wrote the first draft of the manuscript. LR helped performing the experiments and data evaluation. MH helped to draft the manuscript, assisted comparative genomic analyses, and supervised the work of SK. RV initiated the project and supervised the work of SK. All authors read and approved the final manuscript.

## Conflict of Interest

The authors declare that the research was conducted in the absence of any commercial or financial relationships that could be construed as a potential conflict of interest.
