# Supplementary material for: Quantitative Oxygen Consumption and Respiratory Activity of Meat Spoiling Bacteria Upon High Oxygen Modified Atmosphere
Source: Front Microbiol. 2019 Nov 8;10:2398. doi: 10.3389/fmicb.2019.02398 (PMC6857183; doi:10.3389/fmicb.2019.02398)
Supplement: Supplementary file 1 [file Data_Sheet_1.pdf]

Supplementary Material

1 Supplementary figures

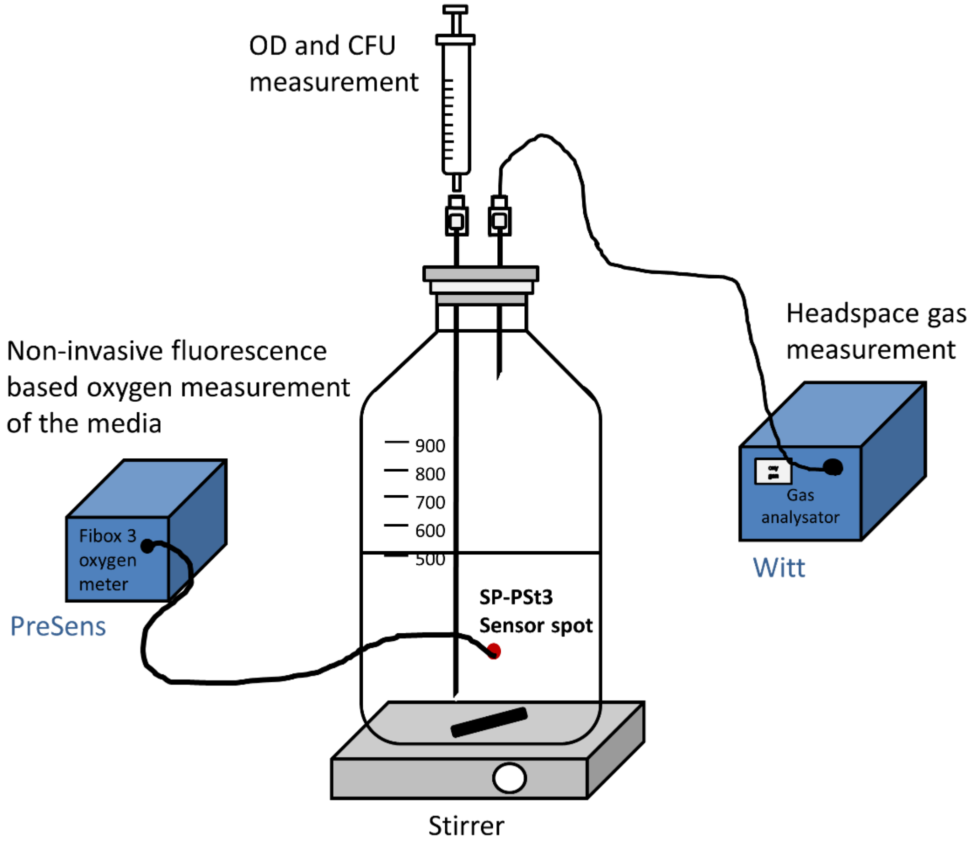

Supplementary Figure S1. Explanation of the experimental setup and measuring methods. Dissolved oxygen of the medium was measured with a non-invasive fluorescence device. The gas composition of the headspace was monitored with a gas analyzer through a sampling cannula. Optical density (OD) and viable cell count was monitored over time, by taking samples with a syringe.

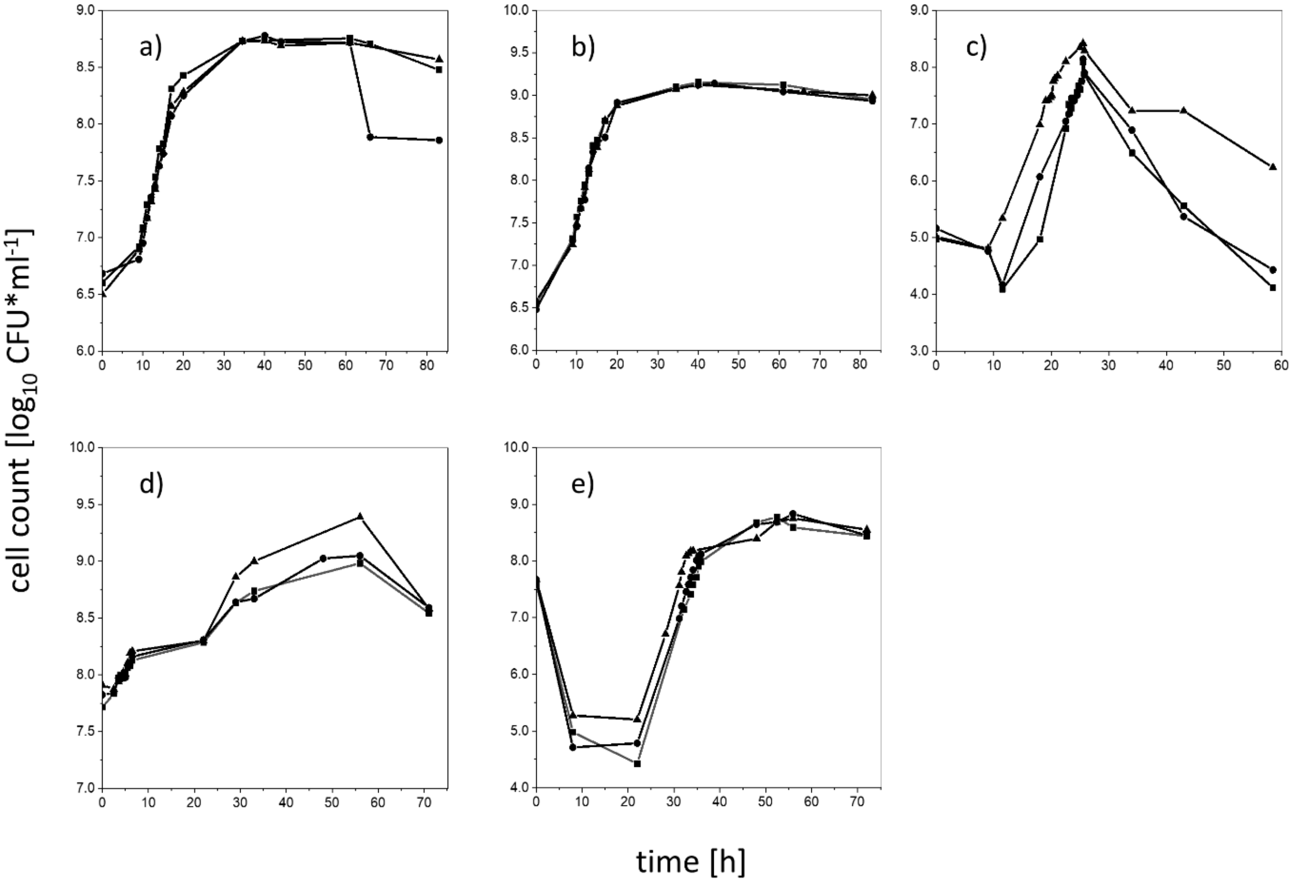

Supplementary Figure S2. Cell growth of all species under high oxygen modified atmosphere. Cell growth curves of a) *L. gelidum* subsp. *gelidum* TMW2.1618 b) *L. gelidum* subsp. *gasicomitatum* TMW2.1619 c) *B. thermosphacta* TMW2.2101 d) *C. maltaromaticum* TMW2.1581 e) *C. divergens* TMW2.1577. Experiments were performed in triplicates (■) replicate1, (●) replicate 2, (▲) replicate 3

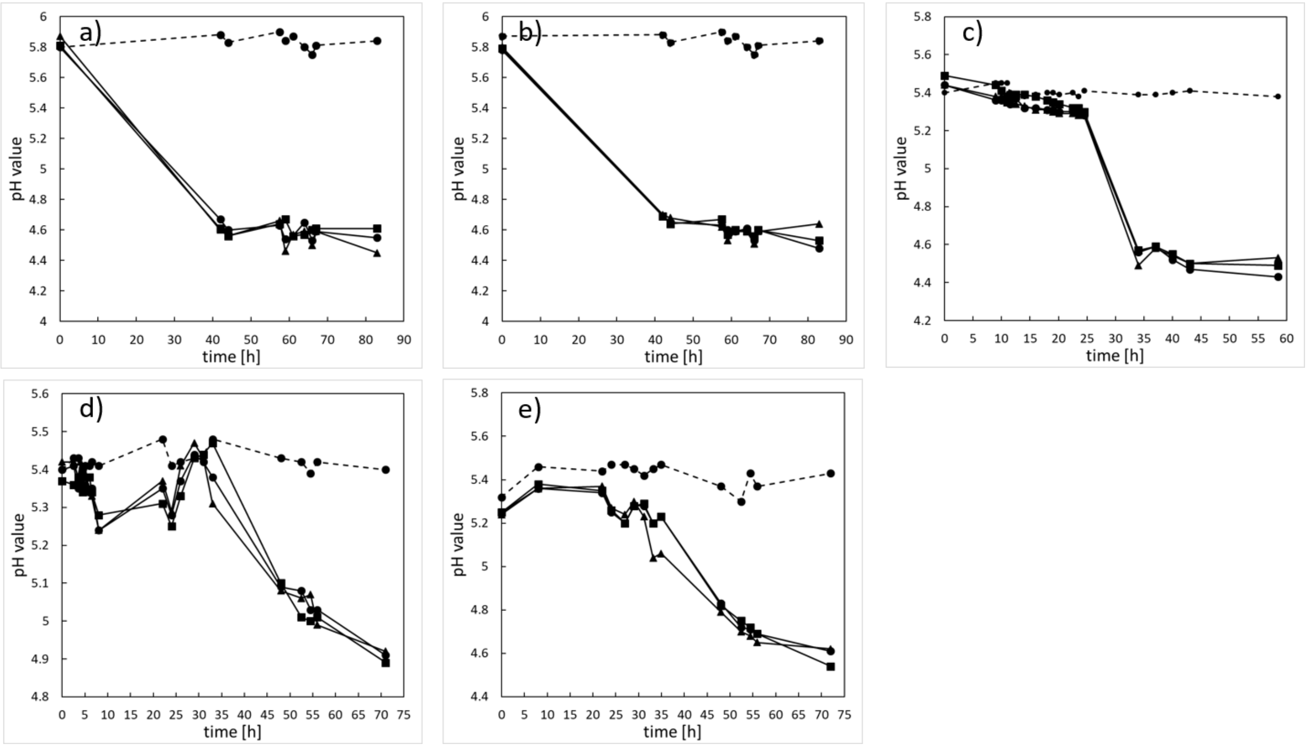

Supplementary Figure S3. pH-evolution of the media cultivated with the species a) *L. gelidum* subsp. *gelidum* TMW2.1618 b) *L. gelidum* subsp. *gasicomitatum* TMW2.1619 c) *B. thermosphacta* TMW2.2101 d) *C. maltaromaticum* TMW2.1581 e) *C. divergens* TMW2.1577. Experiments were performed in triplicates (■) replicate1, (●) replicate 2, (▲) replicate 3 and with a negative control (dotted line).

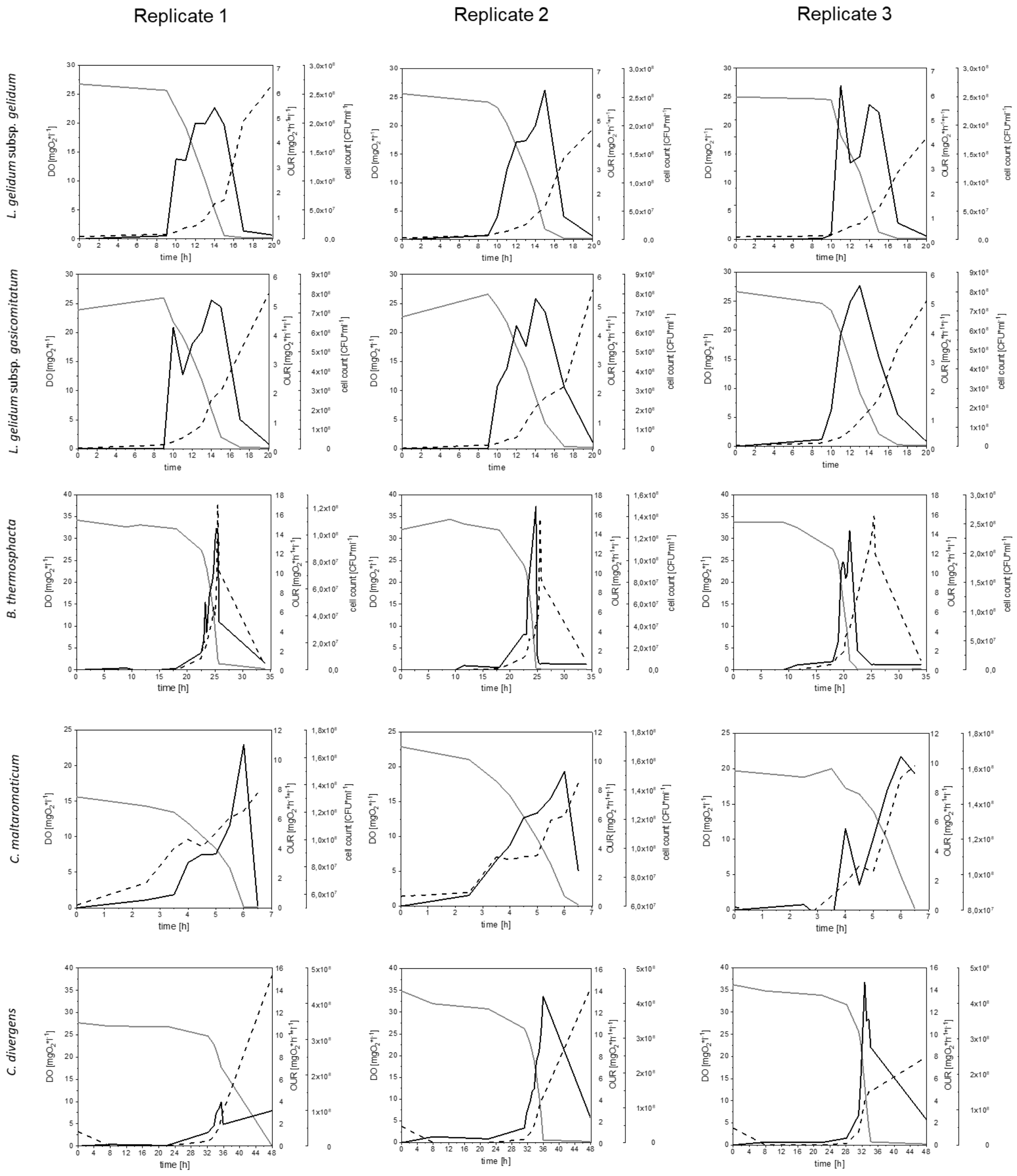

Supplementary Figure S4. Oxygen consumption and cell growth of all species in replicates. Dissolved oxygen [mgO_2*l^{-1}] (grey solid line), oxygen uptake rate [$mgO_2*h^{-1}*l^{-1}$] (black solid line) and cell count [$CFU*ml^{-1}$] (black dotted line) of the five tested spoilage bacteria in replicates.

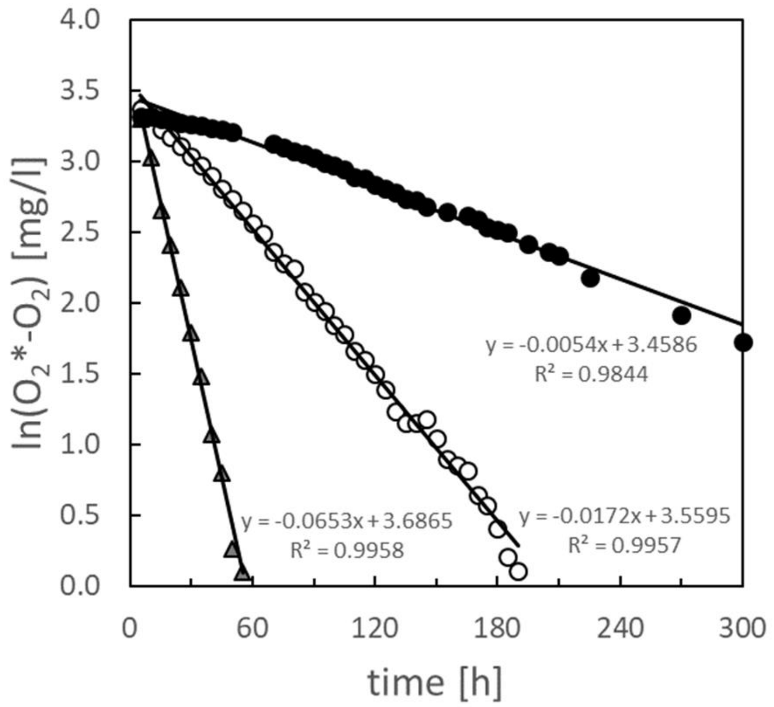

Supplementary Figure S5. Calculation of the volumetric mass-transfer coefficient for each medium. Determination of the volumetric mass-transfer coefficient (K_LA), which is given as the slope of the line (slope=- K_LA). O_2* = oxygen concentration in mg*l^{-1} of oxygen saturated medium. (▲) 12.5 g*l^{-1}, (○) 50 g*l^{-1}, (●) 100 g*l^{-1}.

2 Supplementary tables

Table S1 List of genes needed to establish a citric acid cycle for *B. thermosphacta* TMW2.2101, *L. gelidum* subsp. *gelidum* TMW2.1618, *L. gelidum* subsp. *gasicomitatum* TMW2.1619, *C. maltaromaticum* TMW2.1581 and *C. divergens* TMW2.1577. All predicted gene functions are based on NCBI genome annotation (PGAP) and have been further confirmed by manual BLAST search.

	B. thermosphacta **TMW2.2101**	***L. gelidum*** **subsp.** ***gelidum*** **TMW2.1618**	***L. gelidum*** **subsp.** ***gasicomitatum*** **TMW2.1619**	***C. divergens*** **TMW2.1577**	***C. maltaromaticum*** **TMW2.1581**
**Biosample**	**EHX26**	**BHS02**	**BHS03**	**EH150**	**BFC23**
**Citric acid cycle**					
citrate synthase	07125			03195	05995
aconitate hydratase	04845			03200	04400
isocitrate dehydrogenase	07120			03190	04410
oxoglutarate dehydrogenase	00960<br>01365				
succinyl-coA-synthetase					
succinate dehydrogenase					
fumarate hydratase	01445				03015

malate dehydrogenase					
isocitrate lyase	05055		08230<br>08235<br>08225<br>08220		
